# MIA/CD-RAP Regulates MMP13 and Is a Potential New Disease-Modifying Target for Osteoarthritis Therapy

**DOI:** 10.3390/cells12020229

**Published:** 2023-01-05

**Authors:** Sebastian Staebler, Adrian Lichtblau, Slavyana Gurbiel, Thomas Schubert, Alexander Riechers, Ulrike Rottensteiner-Brandl, Anja Bosserhoff

**Affiliations:** 1 Institute of Biochemistry, Friedrich-Alexander-Universität Erlangen-Nürnberg (FAU), 91052 Erlangen, Germany; 2 Institute of Pathology, Friedrich-Alexander Universität Erlangen-Nürnberg (FAU), Krankenhausstraße 8-10, 91054 Erlangen, Germany; 3 Institute of Applied Pathology, 67346 Speyer, Germany; 4 Institute of Pathology, Medical School, University of Regensburg, 93053 Regensburg, Germany

**Keywords:** osteoarthritis, MIA/CD-RAP, MMP13, therapeutic, signaling

## Abstract

Melanoma inhibitory activity/cartilage-derived retinoicacid-sensitive protein (MIA/CD-RAP) is a protein expressed and secreted by chondrocytes and cartilaginous tissues. MIA/CD-RAP-deficient mice develop milder osteoarthritis than wildtype mice. In this study, we investigated MIA/CD-RAP downstream targets to explain this reduced disease development. As a possible mediator, we could detect matrix metalloproteinase 13 (MMP13), and the influence of MIA/CD-RAP on MMP13 regulation was analyzed in vitro using SW1353 chondrosarcoma cells and primary chondrocytes. The femoral head cartilage of WT and MIA/CD-RAP −/− mice were cultured ex vivo to further investigate MMP13 activity. Finally, osteoarthritis was surgically induced via DMM in C57BL/6 mice, and the animals were treated with an MIA/CD-RAP inhibitory peptide by subcutaneously implanted pellets. MMP13 was regulated by MIA/CD-RAP in SW1353 cells, and MIA/CD-RAP −/− murine chondrocytes showed less expression of MMP13. Further, IL-1β-treated MIA/CD-RAP −/− chondrocytes displayed less MMP13 expression and activity. Additionally, MIA/CD-RAP-deficient ex vivo cultured cartilage explants showed less MMP13 activity as well as reduced cartilage degradation. The mice treated with the MIA/CD-RAP inhibitory peptide showed less osteoarthritis development. Our findings revealed MIA/CD-RAP as a new regulator of MMP13 and highlighted its role as a potential new target for osteoarthritis therapy.

## 1. Introduction

Osteoarthritis (OA) is the most common degenerative joint disorder resulting in the destruction of joint cartilage and underlying bone, along with chronic inflammation of surrounding tissues. Clinical disease management currently involves non-pharmacological interventions and commonly used pharmacological treatments that generally have limited analgesic efficacy and multiple side effects [1]. Disease-modifying osteoarthritis drugs (DMOADs) are pharmaceutical agents to conquer the hallmark of OA pathology, which is reported to be the progressive deterioration in the biological, structural and mechanical properties and function of the joint tissues [2]. With its increasing prevalence and a high burden for public health, there is still an acute need for new disease-modifying targets [3]. The pathogenesis of OA is marked by multiple factors, including mechanical influences, the effects of aging on cartilage matrix composition and structure, as well as genetic factors [4]. OA disease progression is characterized by the degradation of articular cartilage extracellular matrix proteins by the enhanced activity of several matrix metalloproteinases (MMPs) [5]. Among these, MMP13 (collagenase-3) is a major catabolic factor involved in cartilage degradation due to its ability to cleave type II collagen, fibronectin, and small leucine-rich repeat proteoglycans such as fibromodulin [6,7]. The fact that MMP13-knockout mice show a significant inhibition of cartilage structural damage in surgically induced OA confirms that MMP13 activity is crucial for OA development and progression, making MMP13 a promising target for therapeutical intervention [8]. Developing selective efficient MMP13 inhibitors, without unwanted side effects, has already been a challenge in research for years due to the multiple functions of MMP13 and its ubiquitous expression in various tissues [9]. MIA (melanoma inhibitory activity) is an 11 kDa protein expressed and secreted by malignant melanoma and chondrosarcoma cells. Besides tumor cells, MIA/CD-RAP is abundantly expressed and secreted by chondrocytes and cartilaginous tissues and is here referred to as CD-RAP (cartilage-derived retinoic-acid-sensitive protein) [9,10]. MIA/CD-RAP has been established as a marker for chondrogenic differentiation and it is able to induce chondrogenesis via the inhibition of ERK signaling, whereas it inhibits the transition to osteogenic differentiation [11,12]. The absence of MIA/CD-RAP during cartilage development leads to changes in the density, diameter and arrangement of collagen fibers, along with changes in the chondrocytic microvilli number and morphology [13]. During chondrogenesis, the effects of secreted MIA/CD-RAP on the putative transcriptional regulation of additional genes, such as p54nrb via the transcription factor YBX1, have already been elucidated [11]. After OA induction, MIA/CD-RAP-deficient mice develop less severe OA measured by grade and stage of disease progression. Importantly, we could also reveal enhanced chondrocytic regeneration in the MIA/CD-RAP-null mice and an ongoing reduction in OA scores, making MIA/CD-RAP a very interesting target of disease-modifying osteoarthritis drugs [14]. This study characterized and defined MMP13 as a novel MIA/CD-RAP downstream target and investigated the underlying signaling pathways in chondrogenic cells and cartilage. Furthermore, we described the use of an already established MIA/CD-RAP inhibitory peptide, AR71 [15], during osteoarthritis onset and development in vivo. Thus, this current study provides new insights into MMP13 regulation and defines MIA/CD-RAP as a new possible disease-modifying target.

## 2. Materials and Methods

### 2.1. Animal Studies

MIA/CD-RAP-deficient mice and pairing wildtype mice on a C57Bl/6 background were used to obtain primary murine chondrocytes and articular cartilage for in vitro cultivation experiments. MIA/CD-RAP-deficient mice show no harmful phenotype, so no approval was necessary for breeding [13]. Animals were kept under standardized conditions at a temperature of 20–22 °C, relative humidity 46–48% and a 12 h light–dark cycle. Mice were housed in groups of 2–5 per cage and had ad libitum access to water and food.

To determine the effect of MIA inhibitory dodecapeptide AR71 (Ac-FHWRYPLPLPGQ-NH) during osteoarthritis development in vivo, a previously established DMM-osteoarthritis model was used [14]. Briefly, under general anesthesia (i.p. injection of Xylazine (10 mg kg^−1^) and Ketamine (90 mg kg^−1^)), OA was induced in 6-week-old female C57BL/6 mice (Charles Rivers) (*n* = 9 each group) by detaching the medial meniscotibial ligament (DMM) from the tibia plateau. During the same anesthesia, a small incision between the ear and the shoulder was made, and pellets containing the MIA/CD-RAP inhibitory peptide, AR71, or a scrambled peptide (placebo group) were implanted subcutaneously [15]. The peptides were released into the tissue with a continuous release-rate of 2.5 µg per day. Mice were sacrificed 28 days after OA induction, and their knees were dissected, fixed in 4% paraformaldehyde/1×PBS (Carl Roth GmbH, Karlsruhe, Germany), decalcified in 20% ethylene diamine tetraacetic acid (EDTA, Sigma, Taufkirchen, Germany) for 2 weeks and eventually paraffine embedded. Sections (5 μm) were cut on a sliding microtome, stained with hematoxylin and eosin (HE) and Safranin orange and fast green (SO/FG) and the progress of OA was scored by stage and grade by an experienced pathologist (T.S.) using the scoring system proposed by Pritzker et al. [16].

For routine genotyping, genomic DNA was prepared and analyzed by PCR. Whole-genomic DNA extracts were isolated from ear punches using the MyTaq^®^ Extract PCR-Kit (Bioline, Meridian). The primers and PCR conditions used for genotyping are listed and described elsewhere [13]. PCR products were analyzed by agarose gel electrophoresis under UV light.

### 2.2. Cell Culture

SW1353 chondrosarcoma cells were obtained from ATCC ((American Type Culture Collection, Manassas, VA, USA); catalogue number: HTB-94; RRID:CVCL_0543) and cultivated in high-glucose Dulbecco’s Modified Eagle Medium (4.5 g/L glucose; Sigma Aldrich, Seelze, Germany) supplemented with 10% fetal calf serum (FCS, PAN Biotech GmbH, Aidenbach, Germany) and 1% penicillin/streptomycin (PAN Biotech GmbH, Aidenbach, Germany) in an incubator at 37 °C and a humidified atmosphere of 5% CO2 in air. Cells were sub-cultured twice a week at a 1:6 split ratio using trypsin/EDTA solution (0.05%; Invitrogen, Carlsbad, CA, USA). The medium was changed every 48 h. Mycoplasma contamination was excluded on a regular basis. Primary murine chondrocytes were isolated from articular cartilage of four-week-old MIA/CD-RAP-deficient and wildtype mice sharing the same background. Mice were sacrificed by CO2 asphyxiation, and their hip and knee joints were carefully dissected using a stereo microscope under sterile conditions. Articular cartilage was separated from the underlying subchondral bone and digested for 4 h at 37 °C in Collagenase type VIII (Collagenase type VIII from Cl. Histolyticum, Sigma Aldrich, St. Louis, MO, USA; solved in high-glucose DMEM at a concentration of 3 µg/mL). The resulting cell suspension was filtered through a 70 µm cell strainer (Beckton Dickinson, Franklin Lakes, NJ, USA), counted and seeded on polypropylene cell culture flasks (Corning Inc., Corning, NY, USA) at an initial density of 5.000 cells/cm^2^. Maintenance medium consisted of high-glucose DMEM supplemented with 10% FCS, 1% penicillin/streptomycin and 0.5 µg/mL amphotericin B (Sigma Aldrich, St. Louis, MO, USA). Cells were kept in an incubator at 37 °C and 5% CO_2_, detached using trypsin/EDTA solution at 80–90% confluency and split a ratio of 1:3. The medium was changed every 48 h. We used at least 3 different cell isolations per genotype.

### 2.3. High Density Micromass Cultures

To perform three-dimensional re-differentiation of chondrocytes, a modified micromass protocol described by Greco et al. [17] was performed. Briefly, monolayer murine chondrocytes were counted and resuspended in differentiation medium (high-glucose DMEM supplemented with 1% penicillin/streptomycin, amphotericin B, 0.1% ITS premix (insulin 5 µg/mL, transferrin 5 µg/mL, selenious acid 5 ng/mL; Corning Inc.), 0.17 mM L-ascorbic acid (Sigma Aldrich), 0.35 mM L-proline (Sigma Aldrich), 0.1 µM dexamethasone (Sigma Aldrich)) at a density of 2 × 10^5^ cells/mL. To obtain a micromass culture, 20 µL of this cell suspension was pipetted in the middle of a 24-well plate. After an attachment period of 3 h, the wells were carefully flooded with differentiation medium containing 10 ng/mL TGFβ3 (Humanzyme, Chicago, IL, USA) to initiate chondrocytic differentiation. The medium was changed every 2 days. After a re-differentiation period of 7 days, the proinflammatory cytokine interleukin-1 beta (IL-1β) (Humankine, rek. human interleukin-1 beta protein, Biomol, Hamburg, Germany) was added (10 ng/mL) for 24 h. On the next day, the obtained micromass was harvested for RNA extraction, and the supernatant was collected and frozen at −80° for further analyses of MMP13 activity and GAG concentration.

### 2.4. High Density Spheroid Assay

The effect of the MIA-inhibitory peptide AR71 on SW1353 was evaluated in a high-density spheroid assay as described previously [18]. The peptide AR71 (Biochem, Shanghai, China) was dissolved in PBS and had a purity of >95% according to HPLC-MS. Peptide or controls (PBS) were added every day to each spheroid at a final concentration of 10 µM for 2 days, respectively. Eventually, spheroids were subjected to IL-1β or PBS for 24 h. Spheroids were harvested and MMP13 expression was analyzed by qRT-PCR.

### 2.5. siRNA Transfection

A total of 1 × 105 SW1353 cells per well were seeded in 6-well plates and transfected for 72 h with siRNA pool against MIA and YBX or a scrambled siRNA pool (siCTR) (siTOOLs Biotech GmbH, Planegg, Germany), respectively, using the Lipofectamine RNAiMAX transfection reagent (Life Technologies, Darmstadt, Germany). For MMP13 expression and activity analysis, cells were transfected for 48 h and treated with IL-1β (1 ng/mL) for additional 24 h. Cell culture supernatant was collected and frozen at −80 °C for further analyses.

### 2.6. Transfection of Plasmid DNA

Transfection of plasmids into SW1353 cells was performed using Lipofectamine LTX/Plus reagent (Invitrogen). A total of 1 × 10^5^ cells were cultured in 6-well plates and seeded out one day prior to transfection. For transient transfection with expression plasmid, 1 µg of pCMX-PL1-human MIA/CD-RAP plasmid and the empty expression vector pCMX-PL1 were prepared according to the manufacturer’s instructions. The cells were harvested after 24 h of transfection, and total RNA was isolated, as described in Section 2.13.

### 2.7. Luciferase Reporter Gene Assay

For Luciferase reporter assays (LUC) in SW1353, 1 × 105 cells were cultured in 6-well plates, and transfection with plasmid DNA was performed using Lipofectamine LTX/Plus Reagent (Invitrogen) as described above. For each transfection, we co-transfected the human −1042 bp-MMP13 reporter plasmid and the control vector pGL3basic, respectively, with the wild-type Renilla reporter pRL-TK. For harvesting, cells were eventually lysed using 300 µL 1× passive lysis buffer (Promega). Firefly signals were normalized to the corresponding Renilla signals by calculating a Renilla/Firefly ratio and correcting for differences in transfection efficiencies. A Dual-Luciferase^®^ reporter assay (Promega) and a microplate reader (Luc Centro XS^3^ Microplate reader, Berthold Technologies, Bad Wildbad, Germany) were used for measurement. The MMP13 reporter plasmid was generously provided by Ulrike Rottensteiner-Brandl.

### 2.8. Actinomycin Treatment and mRNA Decay

To examine mRNA stability, we treated SW1353 cells with actinomycin D to inhibit the synthesis of new mRNA. Therefore, we transfected SW1353 cells with siPool against MIA/CD-RAP and siCTR, respectively. After 72 h of transfection, 10 µg/mL actinomycin-D was added to each well. Cells were harvested at the beginning of actinomycin D treatment (t0) after 8 h and 16 h. We calculated the half-life of MMP13 mRNA after siPool-mediated MIA/CD-RAP knockdown in comparison to cells transfected with siCTR. For calculation of mRNA half-time, we normalized the expression of MMP13 to the different time points on day 0 (t0).

### 2.9. In Vitro Cultivation and Stimulation of Murine Articular Cartilage Explants

Femoral head cartilage from 4-week-old mice was carefully separated from the underlying subchondral bone using forceps and scissors. Littermates obtained from heterozygous breeding were used to diminish age-specific differences in cartilage development. The obtained cartilage explants were briefly washed in PBS and their wet weight was determined. The explants were then cultured in 24-well plates containing 500 µL of high-glucose DMEM supplemented with 10% FCS, 1% penicillin/streptomycin and amphotericin B (0.5 μg/mL; Sigma-Aldrich Chemie GmbH) for 2 days at 37 °C and 5% CO_2_. After 2 days, the medium was changed to serum-free medium and was equally supplemented. One cartilage explant per mouse was cultivated in the presence of the proinflammatory cytokine IL-1β (10 ng/mL) (Humankine, rec. human interleukin-1 beta protein, Biomol) for 4 days. After the cultural period, the supernatants were collected and stored at −80 °C for further analysis of the MMP13 activity, glycosaminoglycan (GAG) and CTX-II concentration. The cartilage explants were fixed in 4%PFA/1xPBS (Sigma Aldrich) at 4 °C for 24 h and further processed for paraffin embedding.

### 2.10. Histology and TUNEL Staining

For histological analysis, 5 µm paraffin sections were made using a sled microtome. Safranin orange and fast green staining was performed according to the standard protocols. TUNEL staining was performed using the DeadEnd Fluorometric TUNEL System (Promega) according to the manufacturer’s specifications.

### 2.11. DMMB Assay and MMP13 Activity Assay

The concentration of sulfated glycosaminoglycans (GAGs) in cell culture supernatants was measured using a Dimethylmethylene Blue assay (DMMB assay). Duplicates of 20 µL supernatant were transferred into a 96-well plate, and 200 µL of DMMB dye solution was added and extinction was measured at 534 nm. Chondroitin sulfate, a sodium salt from bovine trachea (Sigma Aldrich), was used as an internal standard. Analysis of MMP13 activity was measured using the SensoLyte 520 MMP13 Assay Kit *Fluorimetric* (AnaSpec, Fremont, CA, USA) according to the manufacturer’s instructions.

### 2.12. ELISA

For quantification of cross-linked c-telopeptide of type II collagen (CTX-II) and MIA/CD-RAP in cell culture supernatants, commercially available CTX-II-ELISA (Abbexa, abx153879) and MIA/CD-RAP (Roche) kits were used according to the manufacturer’s specifications.

### 2.13. Analysis of mRNA Expression Using Real-Time PCR

RNA isolation from SW1353 cells was performed using the E.Z.N.A.^®^ Total RNA Kit (Omega Bio-Tek, Norcross, GA, USA) according to manufacturer’s instructions. For RNA isolation from micromass cultures, the E.Z.N.A.^®^ MicroElute Total RNA Kit (Omega Bio-Tek, Norcross, GA, USA) was used. Concentration and purity of the obtained RNA were measured using a NanoDrop device (Peqlab Biotechnologie GmbH). Generation of cDNA was performed as previously described [19]. For real-time PCR, LightCycler^®^ 480 II devices (Roche, Basel, Switzerland) were used with forward and reverse primers from Sigma-Aldrich. Primer sequences are listed in Table 1.

### 2.14. Western Blot Protein Analysis

Total protein isolation and Western blot analysis were performed as described elsewhere [20]. Briefly, 30–100 μg of protein was loaded on 12.75–15% SDS polyacrylamide gels and blotted onto a PVDF membrane (Bio-Rad, Hercules, CA, USA). Blocking was conducted for 1 h using 3% BSA/1xPBS or 5% skimmed milk/1xTBS-T. Primary antibodies against MMP13 (1:6000 in 5% skimmed Milk/TBS-T; Abcam ab39012) and YBX1 (1:500 in 5% skimmed milk/1xTBS-T; Cell Signaling D299) were incubated overnight at 4 °C. For detection of MIA/CD-RAP, we used a previously established antibody (anti-MIA 7638III pure (1:2000 in 3%BSA/1xPBS, Biogenes)) [21]. The primary antibody against β-actin (1:5000 in 5% BSA, Sigma Aldrich, A5441) was incubated for 1 h at room temperature. Horseradish peroxidase-conjugated secondary antibodies (HRP, Cell Signaling 7074 and 7076) were applied for 1 h at room temperature. A Chemostar chemiluminescence imager (Intas, Goettingen, Germany) was used for detection, and LabImage software (Version 4.2.3, Kapelan Bio-Imaging GmbH, Germany) was used for quantification.

### 2.15. Statistical Analysis

Analysis and visualization of experimental results was conducted using GraphPad Prism 9 software (Version 9.1.2. GraphPad Software Inc., San Diego, CA, USA). If not otherwise stated, at least 3 biological replicates were measured. We analyzed experiments with two groups by using two-tailed Student’s *t*-test, while more than two groups were compared by one-way ANOVA followed by Tukey’s HSD post hoc tests. Statistical analysis of Figure 3G, which includes multiple variables, was performed using two-way ANOVA. All results were normalized to the respective control treatment and shown as mean ± SEM, if not otherwise stated. A critical value of *p* < 0.05 was considered statistically significant and is denoted within figure legends.

## 3. Results

### 3.1. MIA/CD-RAP Regulates MMP13

**Figure 1:**
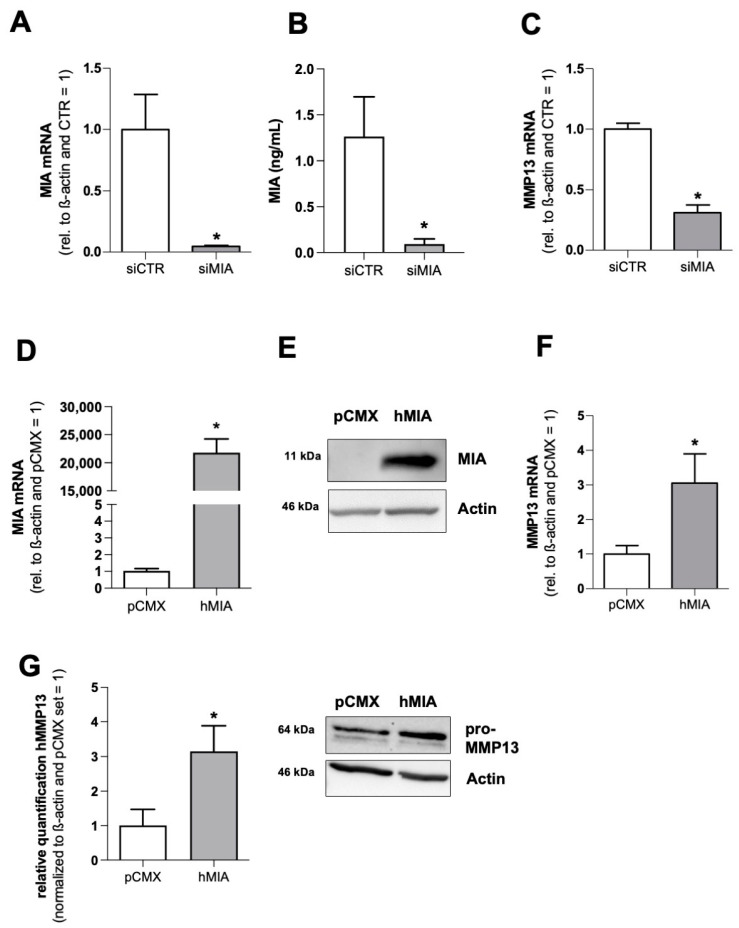
(**A**) Quantitative RT-PCR analysis and validation of MIA/CD-RAP (siMIA) siPOOL-mediated knockdown in comparison to si-Control (siCTR) (*n* = 3). (**B**) Quantification of secreted MIA/CD-RAP protein levels after transfection with siMIA and siCTR by ELISA (*n* = 3). (**C**) Quantitative RT-PCR analysis of MMP13 levels after transfection of siMIA and siCTR (*n* = 3). (**D**) MIA/CD-RAP-mRNA level after transfecting a pCMX_PL1_hMIA-overexpression (OE) plasmid vector and a pCMX_PL1 control vector, respectively (*n* = 5). (**E**) Representative Western blot images depicting MIA/CD-RAP levels after transfection with the OE plasmid and control vector (*n* = 4). (**F**) MMP13 mRNA after transfecting a pCMX_PL1_hMIA-overexpression (OE) plasmid vector (*n* = 5). (**G**) Quantification of MMP13 protein and representative Western blot images depicting pro-MMP13 (64 kDA) protein levels after transfection with the OE plasmid and control vector (*n* = 4). Bars are shown as mean ± SEM (Student’s *t*-test) (*: *p* < 0.05).

To confirm MMP13 regulation by MIA/CD-RAP, we performed an siPOOL-mediated knockdown of MIA/CD-RAP in the human chondrosarcoma cell-line SW1353 (Figure 1A,B) and revealed a significant downregulation of MMP13 mRNA after 72 h of siMIA transfection (Figure 1C). To further evaluate a MIA/CD-RAP-dependent regulation, a MIA/CD-RAP expression plasmid was transfected into SW1353 cells, and expression was validated by qPCR (Figure 1D) and Western blot analysis (Figure 1E). MMP13 was upregulated in the cells transfected with the MIA/CD-RAP expression plasmid (Figure 1F), and higher pro-MMP13 levels (64 kDA) were detected in the Western blot analysis (Figure 1G). These results confirmed the previously described cDNA-array data (Supplementary Figure S1), proving that MMP13 is, in fact, regulated by MIA/CD-RAP [14].

### 3.2. MMP13 Expression and Activity Are Significantly Reduced due to the Loss of MIA/CD-RAP

**Figure 2:**
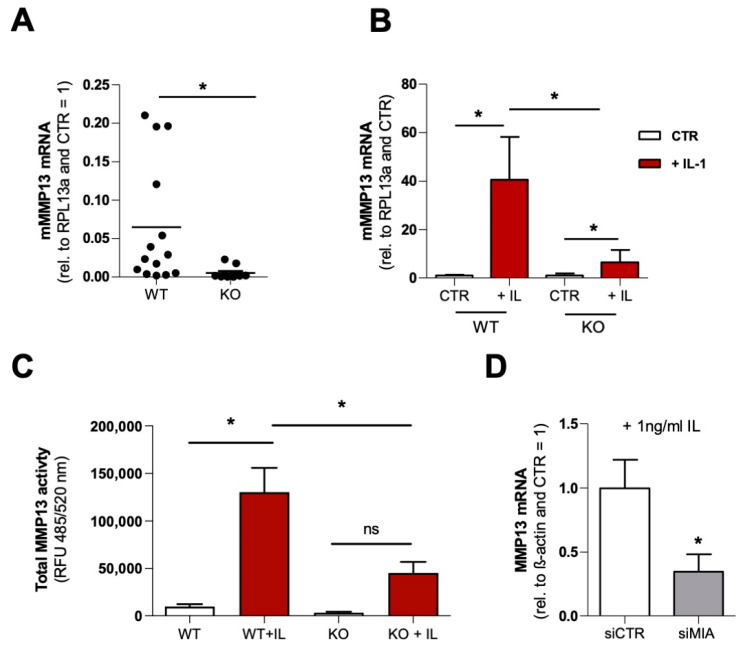
(**A**) Quantitative RT-PCR analysis of MMP13 in murine chondrocytes derived from MIA/CD-RAP-knockout and background-matched wildtype mice; *n* = 14 (WT); *n* = 10 (KO); at least five different isolations per genotype were used. (**B**) Quantitative RT-PCR analysis of micromass cultures stimulated with or without IL-1β for 24 h (*n* = 14 (WT), *n* = 10 (KO); ANOVA followed by Tukey’s HSD post hoc test. (*: *p* < 0.05). (**C**) Total MMP13 activity in micromass culture supernatant after 24 h of stimulation with or without IL-1β (*n* = 9); ANOVA followed by Tukey’s HSD post hoc test (*: *p* < 0.05, ns = not significant). (**D**) Quantitative RT-PCR analysis of MMP13 in SW1353 after transfection with siCTR and siMIA, respectively, following stimulation with 1 ng/mL IL-1β for 24 h (*n* = 5). Bars are shown as mean ± SEM (Student’s *t*-test) (*: *p* < 0.05).

In the next step, we isolated articular cartilage chondrocytes from MIA/CD-RAP-deficient and wildtype C57BL/6-mice. The MIA/CD-RAP-deficient chondrocytes showed significantly reduced expression of MMP13 mRNA compared to the background-matched wildtype chondrocytes (Figure 2A). Given the role of MMP13 as a major driver of osteoarthritis, we wanted to assess the role of MIA/CD-RAP-dependent MMP13 regulation under inflammation. The MIA/CD-RAP-deficient chondrocytes showed significantly reduced expression of MMP13 relative to the wildtype chondrocytes after IL-1β stimulation (Figure 2B). Additionally, the total MMP13 activity in the cell culture supernatants was significantly lower in the MIA/CD-RAP-deficient stimulated chondrocytes compared to the wildtype chondrocytes (Figure 2C). To confirm the regulation during inflammation, siMIA-transfected SW1353 cells were stimulated with IL-1β, and consequently they showed a significantly reduced expression of MMP13 relative to their counterpart siCTR-transfected cells (Figure 2D).

### 3.3. MIA/CD-RAP Regulates MMP13 via YBX1

**Figure 3:**
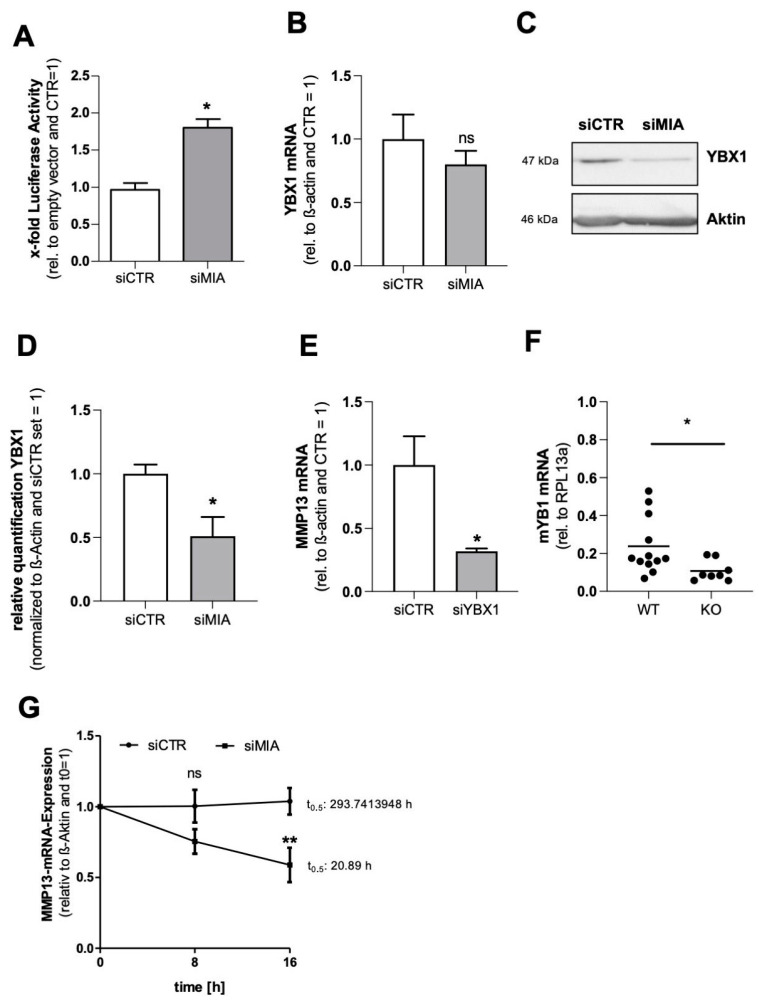
(**A**) MMP13 promoter activity in siCTR- and siMIA-transfected cells (*n* = 5). (**B**) Quantitative RT-PCR analysis of YBX1 after siMIA transfection in comparison to siCTR (*n* = 3). (**C**,**D**) Representative Western blot images and quantification depicting YBX1 protein levels after transfection siMIA (*n* = 4). (**E**) Quantitative RT-PCR analysis of MMP13 levels after siYBX1 transfection in comparison to siCTR (*n* = 4). (**F**) Quantitative RT-PCR analysis of YBX1 expression levels in murine chondrocytes (WT = wildtype, KO = knockout). (**G**) MMP13 mRNA in SW1353 after transfection with siCTR and siMIA, respectively and treatment with 10 µg/mL actinomycin D for 8 and 16 h. MMP13 levels were normalized to β-Actin and an untreated control (*n* = 5) (two-way ANOVA) (**: *p* < 0.005). Bars are shown as mean ± SEM (Student’s *t*-test). (*: *p* < 0.05, ns = not significant).

Since MMP13 expression is mainly regulated on a transcriptional level, we investigated the effect of a MIA/CD-RAP loss on the human MMP13 promoter using a reporter gene assay. As a knockdown of MIA/CD-RAP is supposed to inhibit MMP13 expression, we expected reduced promoter activity. Surprisingly, we found a significant upregulation of MMP13 promoter activity after si-mediated MIA/CD-RAP knockdown using a −1042 bp human MMP13 promoter construct or empty the vector (pCMX_PL1), respectively (Figure 3A). The key to this apparent discrepancy was found after analyzing the role of YBX1, a known downstream mediator of MIA/CD-RAP [22,23]. YBX1 acts as a transcription factor, repressing MMP13 transactivation via the AP-1 site, and on the other hand it acts as an RNA-binding protein. We could determine a significant downregulation of YBX1 protein after MIA/CD-RAP knockdown in the SW1353 cells (Figure 3C,D). To confirm a YBX1-dependent regulation, we performed siRNA-mediated knockdown of YBX1 in SW1353 and confirmed a significant downregulation of MMP13 mRNA (Figure 3E). We confirmed this finding by qPCR analysis of murine MIA/CD-RAP-deficient chondrocytes, which showed significantly less YBX1 mRNA expression compared to the wildtype chondrocytes (Figure 3F). As the promoter activity of MMP13 was not reduced after siRNA-mediated knockdown, we speculated that MIA/CD-RAP regulates MMP13 post-transcriptionally via YBX1. To assess if YBX1 stabilizes MMP13 mRNA, we blocked mRNA synthesis by actinomycin D treatment to determine the rate of MMP13 mRNA decay after silencing MIA/CD-RAP. After MIA/CD-RAP loss, the MMP13 mRNA half-time was 20.9 h in the siMIA-treated versus 293 h in the siCTR-treated cells, proving that MIA/CD-RAP contributed crucially to this high stability of MMP13 mRNA via YBX1 (Figure 3G). Summarizing these data, we showed that YBX1 is a mediator of MIA/CD-RAP-dependent regulation of MMP13 by regulating its mRNA stability.

### 3.4. Decreased Cartilage Degeneration of MIA/CD-RAP −/− Articular Cartilage

**Figure 4:**
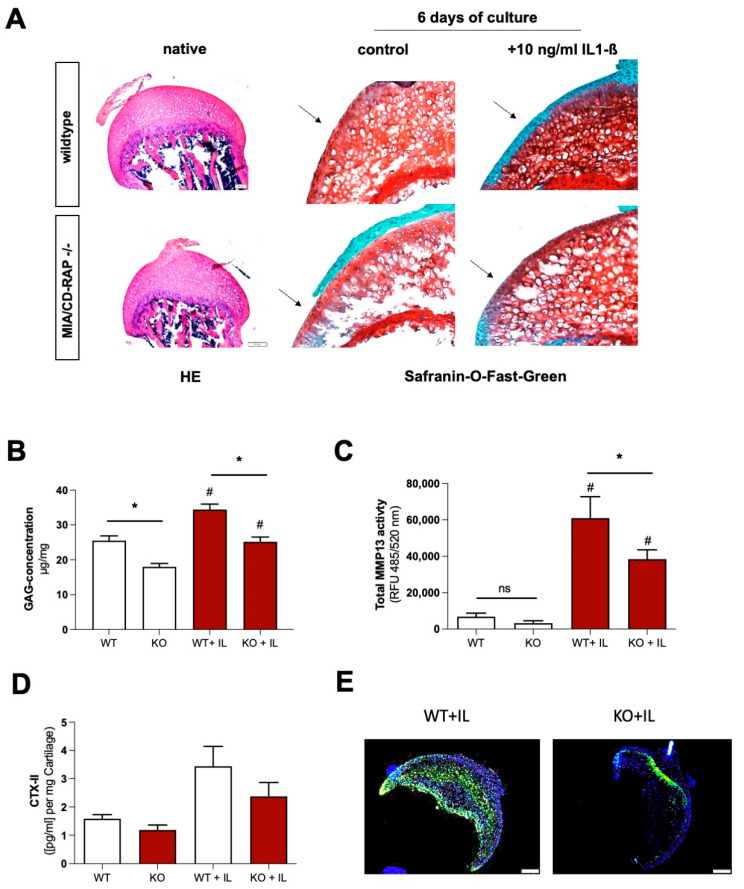
(**A**) Hematoxylin and eosin (HE) staining of wildtype and knockout cartilage. Safranin-O–fast green staining of femoral head cartilage explants after culturing for 4 days with or without 10 ng/mL IL-1β. (**B**) Dimethylmethylene Blue assay (DMMB) to quantify the sulfated GAG release into the medium supernatant (*n* = 10 each genotype). (**C**) Total MMP13 activity in medium supernatant after 4 days of IL-1β. *n* = 10; one-way ANOVA followed by Tukey’s HSD post hoc test; (*: *p* < 0.05.; #: significant (*p* < 0.05) to respective untreated cells) (**D**) ELISA for quantification of c-telopeptide fragments of type II collagen (CTX-II) in medium supernatant *n* = 5; one-way ANOVA followed by Tukey’s HSD post hoc test; *: *p* < 0.05. (**E**) Elevated number of TUNEL-positive cells of in vitro cultured wildtype articular cartilage explants after stimulation with IL-1β. Magnification: 10×; one-way NOVA followed by Tukey’s HSD post hoc test (*: *p* < 0.05). Bars are shown as mean ± SEM.

To investigate the inflammatory degradation of articular cartilage tissue of MIA/CD-RAP-deficient mice, we performed ex-vivo culturing of explanted femoral head cartilage [24]. To imitate an inflammatory setting, we subjected the tissue cultures to IL-1β. HE staining of native articular cartilage did not reveal changes in cartilage size or composition. Safranin-O/fast green staining revealed a higher proteoglycan loss in the wildtype explants subjected to IL-1β compared to the knockout explants (Figure 4A). The quantification of sulfated glycosaminoglycan (sGAG) in medium by DMMB assay showed significantly higher amounts of released sGAG in the untreated and IL-1β-stimulated wildtype cartilage explants (Figure 4B). MMP13 total activity was significantly reduced in the supernatants derived from the MIA/CD-RAP-deficient explants in comparison to the wildtype explants (Figure 4C). Additionally, we analyzed the concentration of cross-linked c-telopeptide of type II collagen (CTX-II) and detected a higher CTX-II release in the wildtype control and IL-1β-treated samples than in the knockout group (Figure 4D). TUNEL staining (Figure 4E) revealed increased cell death in the wildtype cartilage subjected to IL-1β in comparison to the knockout cartilage. Together, these findings suggested that the loss of MIA/CD-RAP led to reduced MMP13 activity, glycosaminoglycan release and apoptosis in ex vivo cultured articular cartilage.

### 3.5. Inhibition of MIA/CD-RAP Reduces OA Development

**Figure 5:**
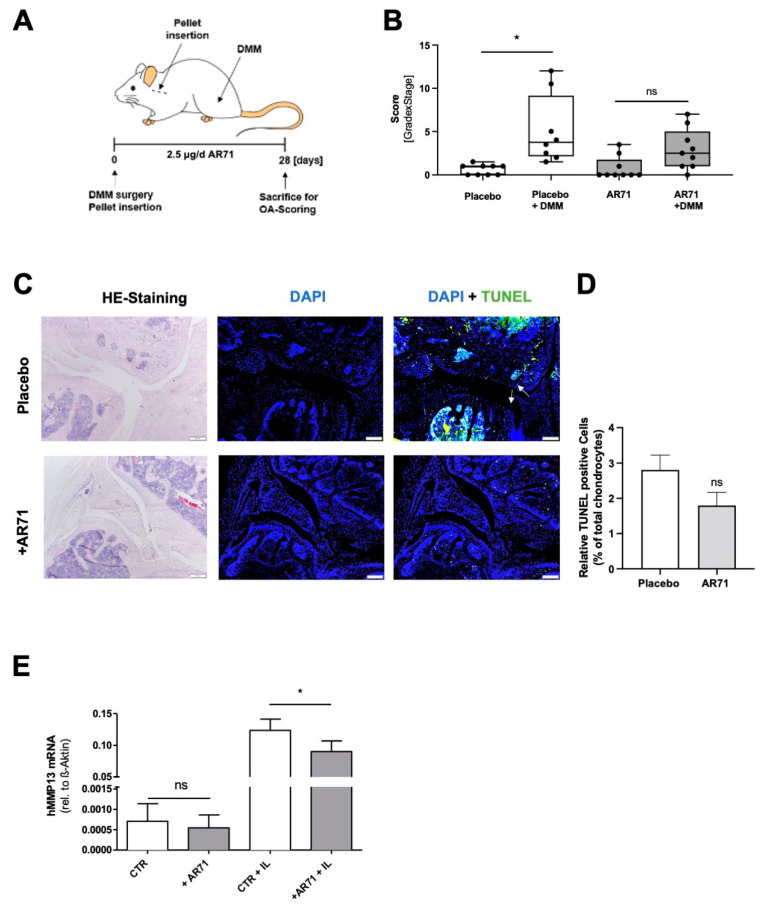
(**A**) Experimental scheme of the conducted animal study. Osteoarthritis was induced by detaching the medial meniscotibial ligament from the tibia plateau of 6-week-old female C57BL/6 mice (black arrow). AR71 was applied by implanting pellets subcutaneously between ear and shoulder of the mice (black line). Implanted pellets without peptide served as a control. Continuous release rate of peptides was 2.5 µg per day. (**B**) OA development was scored by grade and stage 28 days post-surgery; * *p* < 0.05 (one-way ANOVA). (**C**) Hematoxylin and eosin (HE) and TUNEL staining of the medial part of the knee joints after 28 days after OA induction and treatment. (**D**) Quantification of TUNEL-positive cells between placebo and AR71-treated mice. (**E**) Treatment of SW1353 cells with AR71 in a 3D hanging drop spheroid assay. AR71 significantly decreased expression of MMP13 after stimulation with IL-1β (*n* = 3); one-way ANOVA followed by Tukey’s HSD post hoc test (*: *p* < 0.05, ns = not significant). Bars are shown as mean ± SEM.

Finally, we investigated the pharmacological targeting of MIA/CD-RAP as a potential strategy to modulate osteoarthritis onset and development. The dodecapeptide AR71 (sequence: Ac-FHWRYPLPLPGQ-NH2) effectively dissociates MIA–MIA interaction, as previously described [15]. Due to the poor stability and limited permeability through the gastrointestinal tract of peptide therapeutics, we administered AR71 for 28 days by subcutaneously implanting pellets into female C57BL/6 mice and induced osteoarthritis by destabilizing the medial meniscotibial ligament during the same anesthesia (Figure 5A). The untreated mice had significantly increased OA scores after DMM surgery compared to the sham-operated controls. In contrast, the increase in the OA score in the AR71-treated mice was mild, albeit not statistically significant when compared to the sham-operated animals. (Figure 5B). The AR71 treatment also led to a reduction in TUNEL-positive cells in vivo (Figure 5C,D). On a molecular level, treating the SW1353 cells in vitro with 10 µm AR71 for 3 days significantly decreased MMP13 expression under inflammatory conditions (Figure 5E).

## 4. Discussion

In a previous study, we investigated cartilage formation and regeneration in models for osteoarthritis and fracture healing in MIA/CD-RAP-deficient mice in vivo [15]. In that specific study, we revealed an enhanced chondrocytic proliferation in the MIA/CD-RAP-null mice, depicted by enhanced regeneration after osteoarthritis induction. We concluded that proliferating chondrocytes together with their increased matrix production counteract disease progression and therefore promote the regeneration of induced lesions [14]. However, we did not address whether molecular regulations other than enhanced proliferation of mesenchymal stem cells were involved in reduced disease progression. Since the molecular function of MIA/CD-RAP leading to this observation has not been analyzed in detail so far, we aimed to identify the MIA/CD-RAP downstream targets explaining the effect of the reduced OA scoring. 

In the current study, we performed a cDNA array of murine cartilage and found a reduced MMP13 signal in the samples of the MIA/CD-RAP-deficient mice compared to the wildtype samples, indicating a possible MMP13 regulation. Targeted gene silencing of MIA/CD-RAP with small interfering RNAs led to a significant downregulation of MMP13 expression in the chondrosarcoma cell line SW1353. Additionally, the overexpression of MIA/CD-RAP upregulated MMP13 expression. Isolated articular cartilage chondrocytes from the MIA/CD-RAP-deficient mice also showed significantly less expression of MMP13 than the background-matched wildtype chondrocytes. MMP13 is a proteolytic enzyme that belongs to a large family of endopeptidases (matrix metalloproteinases (MMPs)), whose members are essential for extracellular matrix turnover. [25]. Several studies affirmed that MMP13 is associated with articular cartilage destruction, proposing that a high MMP13 expression is associated with cartilage degradation during osteoarthritis [26,27,28]. These results indicated that MMP13 is a critical target during osteoarthritis onset and progression and that MMP13 could be involved in the observed reduced osteoarthritis progression in mice lacking MIA/CD-RAP. These first results in SW1353 chondrosarcoma cells and isolated chondrocytes displayed the regulation of MMP13 by MIA/CD-RAP and were an important baseline for further investigations. 

In our subsequent analysis of the underlying molecular regulation, we surprisingly observed enhanced MMP13 promoter activity after the loss of MIA/CD-RAP. Since gene expression is regulated through transcriptional and post-transcriptional factors, we here proposed a post-transcriptional regulation of MMP13 by MIA/CD-RAP. As the level of abundant mRNA in cells is determined by a balance of transcription and degradation, we here chose to examine the stability of MMP13 mRNA transcripts after a loss of MIA/CD-RAP and inhibition of transcription with Actinomycin-D. It has already been elucidated that MMP13 has a very stable mRNA transcript [29]. Our analysis revealed that a loss of MIA/CD-RAP played a crucial part in stabilizing the MMP13 transcript. As MIA/CD-RAP is not known to possess RNA binding properties, we suspected an indirect regulation of RNA stability mediated by a downstream effector. 

A known MIA/CD-RAP downstream mediator is the Y-box-binding transcription factor 1 (YBX1), which has been described in chondrogenesis and melanoma development [30]. YBX1 belongs to the family of Y-box binding (YB) transcription factors, binding the Y-box promoter element [31]. Besides the role as a transcription factor, YBX1, and especially its cold shock domain W65, has been described as a potent mRNA stabilizer [32]. Although YBX1 binds to the AP-1 site in the MMP13 promoter and represses its expression in HeLA cells [22], shYBX1-silenced A375 melanoma cells had a significant reduction in the expression of MMP13 [33]. Another study revealed a similar regulation of MMP13 by YBX1 in hepatic progenitor cells [34]. Furthermore, MIA/CD-RAP-deficient cells, which express less YBX1, showed reduced MMP13mRNA levels. In the current study, we suggested a possible regulatory effect on MMP13 mRNA by MIA/CD-RAP via YBX1 in differentiated chondrocytes and SW1353 chondrosarcoma cells. Targeting MIA/CD-RAP could lead to a faster MMP13 decay and therefore have a protective effect on cartilage due to reduced MMP13 activity and extracellular matrix cleaving. MIA/CD-RAP-deficient cartilage explants showed significantly less MMP13 activity upon IL-1β stimulation, as measured directly in cell culture supernatants. The potent pro-inflammatory cytokine interleukin-1β (IL-1β) modulates MMP13 expression and is therefore a critical cytokine during osteoarthritis progression [35,36,37]. Here, we wanted to use this modulatory effect to investigate whether MIA/CD-RAP regulates MMP13 even after IL-1β stimulation or whether the stimulation overcomes the reduced stability of MMP13 mRNA. As already established in previous studies, we used a measurement of sulfated glycosaminoglycans in the ex vivo supernatants as a functional correlation for MMP13 activity and feature of cartilage degradation [24]. The MIA/CD-RAP-deficient cartilage explants showed significantly less sGAG and, as a second marker for cartilage degradation, less cross-linked c-telopeptide of type II collagen (CTX-II) [38]. Furthermore, we analyzed apoptosis and revealed less TUNEL-positive cells in the MIA/CD-RAP-deficient explants. A possible explanation for the reduced apoptosis could be that collagen network degradation during osteoarthritis led to changes in chondrocyte anchorage in the cartilage and therefore might have caused chondrocyte apoptosis [39]. Thus, our findings indicated that a loss of MIA/CD-RAP has a protective effect on articular cartilage during osteoarthritis, resulting in reduced cartilage degradation including glycosaminoglycans and collagens and a reduction in apoptosis. Since MIA/CD-RAP is only physiologically expressed in cartilage, it seems to be an attractive therapeutic target for tissue-selective effects [40]. Furthermore, the MIA/CD-RAP-knockout mice developed normally without a macroscopically distinct phenotype, although they had structural abnormalities in the cartilage collagen arrangement seen in electron microscopy [13]. With MIA/CD-RAP, a small, rapidly excreted extracellular molecule, future therapeutics do not have to permeate the cell membrane. A screening phage display for MIA/CD-RAP-binding partners revealed the dodecapeptide AR71 (sequence: Ac-FHWRYPLPLPGQ-NH2) [41]. In a previous study, we revealed that MIA/CD-RAP acts as a homodimer in malignant melanoma [15] and that AR71 successfully dissociates MIA–MIA homodimers in vitro, as measured by a heterogeneous transition-metal-based fluorescence polarization (HTFP) assay [42]. Targeting MIA/CD-RAP dimerization and therefore activity with the inhibitory peptide AR71 has been successfully conducted during melanoma development, and most importantly no adverse effects were observed after an AR71 treatment in mice [16]. In this study, we wanted to assess the effect of this MIA/CD-RAP inhibitory peptide during osteoarthritis development. Since we could reveal a significant reduction in the OA score after 28 days in the previous conducted study with MIA/CD-RAP-deficient mice, we proposed to see a similar effect with the inhibitory peptide. After 28 days of continuous treatment with AR71, we could indeed detect a reduction in the OA score in the AR71-treated mice, albeit not significant. The lack of significance was probably due to a rather mild osteoarthritis development and therefore a high standard variation in the control group. A problem may have been that we started AR71 treatment at the same day as the cartilage destruction occurred, causing a delayed increase in serum levels of AR71, resulting in delayed MMP13 inhibition. Nevertheless, we could also see effects on apoptosis, depicted by the reduced number of TUNEL-positive cells in the AR71-treated mice. Additionally, we could prove that the inhibition of MIA/CD-RAP by AR71 led to significantly reduced MMP13 expression in the treated SW1353 cells after IL-1β stimulation. Generally, unmodified peptides are poor drug candidates due to the fact that peptides get rapidly degraded by proteases in the serum or digestive system. In view of the potentially therapeutic effects of AR71, it seems worthwhile to use chemically modified AR71 or to use AR71 as a lead structure for developing orally available MIA/CD-RAP inhibitors and disease-modifying anti-osteoarthritis drugs. 

In summary, this study suggests that a loss of MIA/CD-RAP protects cartilage degradation during osteoarthritis by regulating MMP13. We could define YBX1 as a downstream mediator of MIA/CD-RAP, which acts as a stabilizer of MMP13 mRNA transcripts. Furthermore, our results underlined that inhibiting MIA/CD-RAP is a promising strategy in the search for new potential disease-modifying targets.

## Figures and Tables

**Table 1:** Oligonucleotides used for real-time PCR.

Gene	Forward Primer	Reverse Primer
ACTB (human)	CTACGTCGCCCTGGACTTCGAGC	GATGGAGCCGCCGATCCACACGG
MMP13 (human)	TACCAGACTTCACGATGGCATTGCTG	AAAGTGGCTTTTGCCGGTGTAGGTG
YBX1 (human)	GGGACAAGAAGGTCATCGCA	GTAACATTTGCTGCCTCCGC
MIA (human)	CATGCATGCGGTCCTATGCCCAAGCTG	GATAAGCTTTCACTGGCAGTAGAAATC
RPL13a (murine)	CCGGAAGCGGATGAATACCA	ACAACCTTGAGAGCAGCAGG
YBX1 (murine)	ATCGCAACGAAGGTTTTGGG	GTAACATTTGCTGCCTCCGC
MMP13 (murine)	CAGGAGCCCTGATGTTTCCC	TCCTCGGAGACTGGTAATGG

## Data Availability

Not applicable.

## Supplementary Materials

The following supporting information can be downloaded at: https://www.mdpi.com/article/10.3390/cells12020229/s1, Figure S1: cDNA-Array—ACT, MIA1, MMP13 signals.
