# Supplementary material for: MIA/CD-RAP Regulates MMP13 and Is a Potential New Disease-Modifying Target for Osteoarthritis Therapy
Source: Cells. 2023 Jan 5;12(2):229. doi: 10.3390/cells12020229 (PMC9856983; doi:10.3390/cells12020229)
Supplement: Supplementary file 1 [file cells-12-00229-s001.zip › cells-2122705-supplementary.pdf]

Supplementary Data

Figure S1: cDNA-Array – ACTB, MIA1, MMP13 signals

Gene Title	Gene symbol	MIA KO 1	MIA KO 2	wildtype	wildtype
actin beta	actb	48574,84	18671,47	28436,93	16787,84
melanoma inhibitory activity 1	MIA1	54.20	3.81	1116.22	393.50
matrix metallopeptidase 13	MMP13	320.91	119.93	1178.43	1957.16
